# Single case-control design for the study of the neuropsychological deficits and dissociations in Huntington’s disease-like 2

**DOI:** 10.1016/j.mex.2020.100782

**Published:** 2020-01-10

**Authors:** Aline Ferreira-Correia, David G. Anderson, Kate Cockcroft, Amanda Krause

**Affiliations:** a University of the Witwatersrand, Department of Psychology, School of Human and Community Development, Johannesburg, South Africa; b University of the Witwatersrand Donald Gordon Medical Centre, Neurology, Johannesburg, South Africa; c Division of Human Genetics, National Health Laboratory Service and School of Pathology, Faculty of Health Sciences, University of the Witwatersrand, Johannesburg, South Africa

**Keywords:** The Single-Case Methodology in Neuropsychology (Crawford & Howell, 1998), HDL2, Huntington’s disease, Study case, Neuropsychology, Neuropsychological assessment

## Abstract

The Single-Case Methodology in Neuropsychology (Crawford & Howell, 1998) is a research design and robust inferential statistical method that facilitates the neuropsychological description of one case in terms of the differences between its profile and the performance of a carefully matched sample (Crawford & Garthwaite, 2012). The comparison is made by means of a *t*-test statistic that treats the normative sample as a sample and not as a population, with a particular effect-size associated with the size (n) of the sample. It is an ideal method for the neuropsychological investigation of rare diseases, such as Huntington’s Disease Like-2 (HDL2), especially when the cases are embedded in contexts of great diversity. This paper presents a step by step guide to the implementation of this method in a series of demographically and clinically diverse group of patients.

• The application of a Single-Case Methodology in Neuropsychology enables the characterisation of rare diseases while controlling for demographic and context-related variables.
• The implementation Single-Case Methodology in Neuropsychology provides test norms for homogenous groups that can be used by practitioners in their clinical work.
• The method was customised for the South African population by controlling variables of specific relevance, such as linguistic diversity and quality of education.

## Graphical abstract

**Figure:**
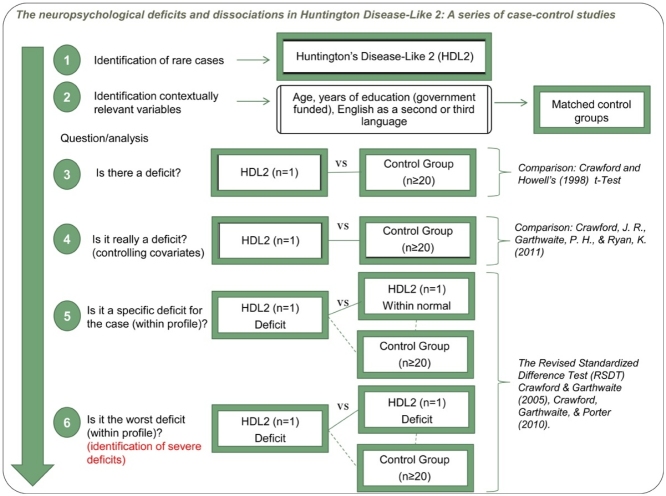

**Specification Table**

**Table:** 

Subject Area:	Psychology
More specific subject area:	Neuropsychology
Method name:	The Single-Case Methodology in Neuropsychology [1]
Name and reference of original method:	Crawford, J. R., & Garthwaite, P. H. (2007). Comparison of a single case to a control or normative sample in neuropsychology: Development of a Bayesian approach. Cognitive Neuropsychology, 24(4), 343-372. doi: 10.1080/02643290701290146 Crawford, J. R., Garthwaite, P. H., Azzalini, A., Howell, D. C., & Laws, K. R. (2006). Testing for a deficit in single-case studies: Effects of departures from normality. Neuropsychologia, 44(4), 666-677. doi: 10.1016/j.neuropsychologia.2005.06.001 Crawford, J. R., Garthwaite, P. H., & Gray, C. D. (2003). Wanted: Fully operational definitions of dissociations in single-case studies. Cortex, 39(2), 357-370. doi: 10.1016/S0010-9452(08)70117-5 Crawford, J. R., Garthwaite, P. H., & Ryan, K. (2011). Comparing a single case to a control sample: Testing for neuropsychological deficits and dissociations in the presence of covariates. Cortex, 47(10), 1166-1178. doi: 10.1016/j.cortex.2011.02.017 Crawford, J. R., Garthwaite, P. H., & Wood, L. T. (2010). Inferential methods for comparing two single cases. Cognitive Neuropsychology, 27(5), 377-400. doi: 10.1080/02643294.2011.559158 Crawford, J. R., & Howell, D. C. (1998). Comparing an Individual's Test Score Against Norms Derived from Small Samples. The Clinical Neuropsychologist, 12(4), 482-486. doi: 10.1076/clin.12.4.482.7241
Resource availability:	All programs used for the single case analyses were downloaded from Professor John Crawford’s personal page hosted by the University of Aberdeen (http://homepages.abdn.ac.uk/j.crawford/pages/dept/psychom.htm).

## Method details

### Step 1: Assessment of the suitability of the method

#### Identification of rare cases

The Single-Case Methodology in Neuropsychology [1] is a research design and robust inferential statistical method that facilitates the neuropsychological description of one case in terms of the differences between its profile and the performance of a carefully matched sample of modest size [2]. Thus, it is ideal to implement for rare or unique cases or when the establishment of clinical groups is not possible due to the heterogeneity of the cases in terms of relevant clinical variables (e.g. disease progression, age of onset, and severity of the genetic mutation).

Huntington Disease Like-2 (HDL2) is an autosomal dominant genetic disease caused by mutations that produce a pathogenic expansion of a triplet repeat in chromosome 16q24.3 in a variably spliced exon of junctophilin-3 (JPH3). It is the phenocopy that most resembles the clinical presentation of Huntington’s Disease (HD). Huntington’s Disease Like-2 is very rare [3,4], with a strong ethnic implication as most, if not all, of the individuals with HDL2 have predominantly African ancestry [51](Rudnicki & Margolis, 2008). Consequently, no group studies on the characterisation of HDL2 are available.

#### Identification of context-relevant variables

This method performs a comparison between the case’s scores and the mean of the matched control group. This comparison is made by means of a *t*-test statistic that treats the normative sample as a sample and not as a population (unlike *z* scores), with a particular effect-size associated with the size (*n)* of the sample. A premise of the single-case study methodology is that the matched control sample potentially represents the premorbid functioning of the clinical case, thus, it is important to identify relevant contextual variables that act as a moderators of cognitive performance. Specifically, the selection of a matched control sample permits the investigation of neurocognitive performance while indirectly controlling for covariates [1,[5], [6], [7], [8]], such as age, level of education [[9], [10], [11]], quality of education [12], and ethnicity (in terms of linguistic practices and historical disadvantages attached to ethnicity) [13,14], which have a significant impact on cognitive testing.

In South Africa, neuropsychological assessment faces great challenges linked to the history of apartheid which resulted in significant socioeconomic inequalities and variances of educational opportunities associated to ethnicity [15], as well as vast linguistic and cultural diversity [16]. These factors are associated with wide performance differences between standardised tests scores of different samples, especially when South Africans are compared against foreign norms [17]. The most affected by this bias are those South Africans from rural areas and/or who attended disadvantaged schools [18,19]. Therefore, the selection of matched control samples was conducted in order to control for the following context-relevant variables: age, level of education, type of school (public/government only), English second language speakers (polyglot participants only), and race. All participants in the study self-identified as belonging to two racial groups that have a history of socioeconomic disadvantage in South Africa (Black and Mixed race^1^).

Other variables, such as developmental history [21,22], gender [10], current pharmacological treatment [23], socioeconomic status [24], medical factors linked to HD/HDL2 (such as repeat length and age of onset) [25], acculturation [12], fatigue [26], mood [27,28], motivation [29], testing environment [26], and test wiseness [30], are moderators, to different degrees, of test performance. While these variables could not be strictly controlled, it is anticipated that their effects were mitigated by matching the controls and the HD patients to the HDL2 patients. Nevertheless, the possibility of their effect on the performance on the test must be borne in mind. Participants with medical histories with a known impact on cognition, such as HIV, metabolic and other neurological conditions (e.g. traumatic brain injury), were excluded from the sample.

#### Sampling considerations

A total population sampling method [31] was implemented for the recruitment of the HDL2 cases whereas a purposive homogenous sampling [31] was used for the configuration of the healthy control groups. The healthy participants were matched to the clinical cases in terms of age, years of education, type of schooling (only public, primary and secondary schools were included), and ethnicity. The healthy controls did not have a history of genetic disease, or any reported motor, psychiatric or cognitive symptoms. Only participants who were able to converse in English were included from the clinical and healthy sample, while those participants with other comorbid neurological or metabolic diseases, a history of traumatic brain injury with loss of consciousness, abuse of illegal drugs, and/or who did not give formal consent, were excluded from participation.

The research design and the statistical method linked to it is suitable even for very small control samples (n = 5), nevertheless, predictive power is associated with the size of the control sample, with bigger samples possessing greater power to detect differences and effects [1,8]. A sample of at least 20 controls was the minimum required to reject the null hypothesis and to reduce the rate of type I error [32]. Therefore, the size of the control groups met the criterion of *n*≥20.

### Step 2: Establishment of the assessment protocol

Since the purpose of the research was to establish a cognitive profile of HDL2 (deficits and dissociations), measurements of a wide array of constructs in both visual and verbal domains were included. Subsequently, test selection was guided by three aspects: the identification of instruments frequently used in the Huntington’s Disease (HD) literature (given the similarities between HDL2 and HD), availability of the instruments and the time constraints imposed by the study design. Given that participation in the study involved other activities besides the neurocognitive assessment, only two hours were available for the evaluation of each patient, so preference was given to brief tests. All the instruments used are described below.

The *Demographic questionnaire* assessed variables such as: age, level of education, occupation, language experience (language spoken in order of self-assessed proficiency and languages used daily), gender, ancestry, HDL2 history (diagnosis, onset and symptomatology), general medical history and medication.

The *Montreal Cognitive Assessment (MoCA)* [33] is a neuropsychological screening test for identifying cognitive impairment. It assesses a variety of cognitive functions, including attention and concentration, executive functions, memory, language, visuoconstructive abilities, abstract thinking, arithmetic ability and orientation in approximate 10 min [33]. The English 7.1 version was used (Lion, Rhinoceros, Camel, Version). The total score was calculated including the additional point awarded to the participants with less than 12 years of education.

The *Rey Auditory Verbal Learning Test (RAVLT)* [34] assessed verbal attention and concentration span under overloaded conditions [26], and evaluates verbal memory (immediate, delay and recognition). It takes approximately 20 min to be administered and the lists A–B were used.

The *Stroop Word-Colour Interference Test (SWCIT)* [35] measures concentration (selective attention) and executive function (cognitive flexibility and inhibition) [35]. This test consists of three pages with 100 items (of words Red, Green and Blue) each organised in five columns of 20 items; the first page (Word Reading) presents only the words in black ink organised randomly, the second page (Colour Naming) consists of the symbols XXXX printed in either of the three inks and, on the fourth page (Colour-Word Reading), pages one and two are combined, having a word printed in a different colour than the name suggests [35]. The participant has only 45 s for each trial, hence the test takes approximately 5 min to administer.

Two subtests from the Wechsler Adult Intelligence Scale-IV (WAIS-IV) were selected, including the *Symbol Search Subtest* [36], which evaluates visual attention in terms of information processing speed and visual perception [36]. In this subtest, the examinee must mark ‘yes’ or ‘no’ if one of the target symbols is found in a group of symbols. The participant should correctly identify as many items as possible within 120 s [36]. The *Letter-Number Sequencing Subtest* [36] assesses verbal working memory. Here, the participant must rearrange a combination of numbers and letters that are presented to them orally. It consists of 7 items with 3 trials each and the test has a duration of 10 min approximately.

From the Wechsler Memory Scale-IV (WMS-IV) two subtests were included in the assessment battery. Firstly, the *Spatial Addition Sub-Test* [37] asseses visual working memory by asking the participants to solve a problem of visual addition presented in a sequence of grids and following a set of rules [37]. Secondly, the *Visual Reproduction I and II Subtests* [37] evaluate immediate (I) and delayed (II) non-verbal memory. For Visual Reproduction I, the participant must draw a design from memory after viewing it for 10 s. For Visual Reproduction II, the participant must draw the same designs from the first trial after a period of interference and is also required to choose from six designs those that matched the original ones (immediate recall).

The *Controlled Oral-Word Association Test (COWAT)* [38] is a measure of verbal fluency, which consists of three naming trials (frequently the letters F, A and S [26]. The participant is asked to say as many words as possible starting with each letter, for one minute per trial [38].

Two subtests were selected from the Delis-Kaplan Executive Function System (D-KEFS). Firstly, the *Design Fluency Test* [39], which consists of an unstructured activity in which the participant is required to produce, in three trials of 60 s each, as many different designs as possible, by connecting dots in a grid using a pencil. It assesses non-verbal fluency, cognitive flexibility and response inhibition [39]. Secondly, the *Tower Test* [39] was selected from the D-KEFS. The purpose of the task is to build a designated or target tower in the fewest number of movements possible using discs varying in sizes (small to large), following two rules: move only one disc at a time and never place a larger disc over a smaller disc [39].

The *Symbol Digit Modalities Test (SDMT)* [40] assesses divided attention, visual scanning, tracking and motor speed [41]. It consists of a substitution task where the participant has to pair numbers with geometric symbols for 90 s [40], which is done in writing for the first form, and orally in the second form.

The *Hooper Visual Organization Test (HVOT)* [42] measures visual organization ability. It consists of 30 pictures of cut up objects that have been rearranged and the examinee has to recognise and name each [26].

Lastly, the *Purdue Grooved Pegboard Test (PGPT)* [43] assesses manual dexterity [26]. The objective of the task is to place pegs, one by one, as quickly as possible, in a row of holes, using firstly the dominant hand and, in the second trial, the non-dominant hand [41].

The Magnetic Resonance Imaging (MRI) conducted on the HDL2 patients was done at the University of the Witwatersrand Donald Gordon Medical Centre Radiology Department on a 1.5 T Philips Intera MR scanner (Philips Medical Systems, Eindhoven).

All clinical participants met with an independent genetic counsellor who provided detailed information about the study and obtained formal written consent. The consent from the healthy participants was obtained directly by the assessor. The implementation of the research protocol after consent included, first, the neurological assessment, which culminated with the procurement of a blood sample. Second, the participant undertook the neuropsychological assessment. Third, the participant was transported to the MRI facilities, where lunch was provided upon arrival. Variations of this typical protocol were sometimes necessary in order to accommodate for special circumstances such as refusal or inability to take part in certain aspects of the study (e.g. MRI or neuropsychological assessment).

The order of administration was as follows: 1) Demographic questionnaire, 2) MoCA, 3) RAVLT (trials I to V, trial B, trial VI), 4) Design Fluency Sub-Test, 5) SDMT, 6) HVOT, 7) Spatial Addition Sub-Test, 8) RAVLT Delayed trial and Recognition Trial, 9) Visual Reproduction I Sub-Test, 10) Letter-Number Sequencing Sub-Test, 11) Symbol Search Sub-Test, 12) COWAT, 13) Tower Sub-Test, 14) SWCIT, 15), Visual Reproduction II Sub-Test, and 16) PPT. Behavioral and clinical notes were kept across the entire assessment. Variations on this protocol took place due to issues beyond the researchers’ control; nevertheless, the interruptions were managed in order to preserve the delay periods in the memory tests. Breaks were offered after the RAVLT recognition trial. All assessments were conducted in a well-lit and ventilated space, on a flat surface (table or desk) in a quiet room.

In order to establish the neuropsychological profile of HDL2, it was necessary to identify the pattern of multiple cognitive functions. Although no single test provides a pure measure of a particular function, for the purpose of facilitating the interpretation of the profile, specific tests or subtests have been grouped under the core cognitive domain that each test intends to assess. Thus, the specific core tests assessing each of the cognitive variables are specified in Table 1.

**Table 1:** Operational definition for each cognitive function to be assessed.

Function/Modality		Test
Attention and concentration		Rey Auditory Verbal Learning Test (trials I and B) Stroop Word Colour Interference Test (SWCIT) trials 1 (Word) and 2 (Colour)
Working memory	Verbal	Letter-Number Sequencing (LNS Total WAIS-IV and LNS Span WAIS-IV)
	Non-verbal	Spatial Addition (Wechsler Memory Scale-IV [WMS-IV])
Memory and learning	Recent Verbal	Rey Auditory Verbal Learning Test ([RAVLT] I to V)
	Recent Non-verbal	Visual Reproduction I (WMS-IV)
	Delay Verbal	RAVLT (delayed trial)
	Delay Non-verbal	Visual Reproduction II (WMS-IV)
	Recognition Verbal	RAVLT (recognition trial)
	Recognition Non-verbal	Visual Reproduction (recognition)
Executive functioning	Verbal fluency	Controlled Oral Association Test
	Non-verbal fluency	Design Fluency (Delis-Kaplan Executive Function System [D-KEFS]), Conditions 1, 2 and 3.
	Planning	Tower Test (Delis-Kaplan Executive Function System [D-KEFS])
	Inhibition	SWCIT (Colour/Word Trial)
Psychomotor speed and dexterity		Symbol Digit Modalities Test (SDMT) Purdue Grooved Pegboard Test (PGPT)
Visuo-constructive ability		Hooper Visual Organization Test (HVTO)

### Step 3: Preliminary analyses

#### Normality assessment

Crawford and Howell’s [1] *t-*Test requires the assumption of a normal distribution to be met. Therefore, the characteristics of the distribution were reviewed by means of visual inspections of data histograms for each control group separately, using SAS version 9.4 [44] (Supplementary Data File 1). For sample groups of 30 or fewer, the use of histograms is recommended to assess normality [45]. Most of the variables appeared to be normally distributed with slight variations in terms of kurtosis and skewness. However, this did not hold for extreme cases, which were found in the following tasks: RAVLT Recognition trial, and SDMT Speed and Accuracy Index; thus affecting the conditional distribution and increasing the possibility of Type I error. Ceiling effects are common in tasks that should be within the competence of healthy controls and in measurements of pathological deviations from the instruction (e.g. errors), which cause an extreme distribution that is skewed and most likely leptokurtic [32]. According to the argument and empirical evidence presented by Crawford et al. [32], this is highly problematic for smaller samples (n≤20) where the risk of Type I error increases considerably. Bearing in mind that all control samples in the current study comprised approximately 20 participants, the limitation of small samples may apply to this study. Yet, methods available to normalise the data are not applicable for single case control studies where the samples are too small to adjust, or when the characteristics of the test do not allow for such transformation given the limited range of scores [32]. Both conditions are applicable to the data from the control groups’ data in this research.

Nonetheless, Crawford et al. [32] still recommend that the method be used on the grounds of sustaining its power when the assumption of normality is not met [1], even under extreme conditions of kurtosis and skewed distributions [32]. In addition, *t-*scores are significantly less biased than *z*-scores and can provide a reliable measure of the probability of the score’s deviation from normality. In spite of its robustness, the authors warn about the increased risk of incorrectly judging a score as deficient due to an inflation of Type I error rate. Given these guidelines and the exploratory nature of the study, only the tasks with a standard deviation of zero (such as Number of Pegs achieved at five minutes in the PGPT) and those that were extremely skewed (such as Orientation of the MoCA) were excluded. Other tasks which displayed a non-normal distribution were not excluded from the analysis of deficit, these findings were interpreted cautiously and the associated risks and limitations of this decision are highlighted in the Discussion chapter.

#### Correlations

The modified *t*-test analysis [1,46] allows for the exploration of deficits while controlling for the effects of covariates, thus, correlational analyses were necessary. Age and level of education are the most important covariates of neuropsychological performance [7]. Although the controls were matched on these two variables, meaningful variations within the parameters (in a somewhat arbitrary manner) are possible. A third variable that correlates highly with cognitive performance is psychomotor speed [7,47]. Since this is a function severely affected in HDL2, it is important to determine if the deficit in an ‘unrelated' function was moderated by psychomotor speed. Three measures were considered specifically for the purpose of exploring potential psychomotor speed effects: PGPT time dominant, PGPT time non-dominant, and Symbol Search Correct. These were chosen because they displayed distributions that appeared normally distributed and are reliable measures of speed. As for age and years of education, if any of these covariates significantly correlated with the task under investigation, new analyses were conducted in order to control for their possible moderation effects. If more than one of the speed of processing tests correlated significantly with the task at hand, the one with the highest and most significant correlation was chosen for the analysis.

It is important to note that this method does not require a multivariate or bivariate normal distribution [7]. Therefore, a correlation matrix was carried out using SPSS version 9.4 for Windows [48] and it was conducted separately for each control group (Supplementary Data File 2). Specifically, this analysis consisted of pairwise correlations between variables, which were measured by Spearman’s correlation coefficient. The selection of Spearman’s correlation over Pearson’s is justified by the small sample size and the histograms, which do not show normal distributions for most variables.

#### Data selection

The initial analysis were conducted using 64 test variables, which included, for example, all the subtests of the MoCA and all the errors and times on the different tests. This number was reduced to 33 after implementing a “data reduction strategy” (Stout et al., 2012, p. 5) that involved selecting the tests or subcomponents thereof that are known to have the best psychometric properties, especially in terms of their sensitivity to measure the cognitive variable of interest. This was a necessary step as all the calculations are done manually and one by one.

### Step 4: Analysis of deficits

The analysis of deficit was achieved by comparing the raw score^2^ of the patient on each of the tests with the performance of the matched control group. The *t*-test developed by Crawford and Howell [1], based on Sokal and Rohlf’s method (1995, as cited in Crawford and Howell [1]), was the inferential statistical method used to explore the presence of deficits in all cases. This method tests whether the score obtained in a test is significantly below that of the control. A significant difference is therefore interpreted as deficit, thus, both of these terms indicate extremely low performance. The method was further updated by Crawford et al. [46], and the newer version provides additional data regarding the percentage of the control population that would obtain a score lower than the patient, as well as estimates of effect sizes.

The operational definitions provided by Crawford et al. [6] for interpreting a score are:

- Within normal limits: the patient’s score on a given test is not significantly lower than that of controls using Crawford and Howell [1] and Crawford et al. [7] method and a one-tailed test (p < 0.05).
- Deficit: the patient’s score on a given test is significantly lower than that of controls using Crawford and Howell [1] and Crawford et al. [7] method and a one-tailed test (p < 0.05).

The severity of deficit can be inferred from the size of the *t*-test in conjunction with the *p*-value. A large *t*-test would be indicative of a large distance between the case’s score and the control mean, which, if negative, would indicate a severe deficit, similarly to the interpretation of the *z*-score. Considerations regarding the distribution of the control group should be included in this reading of severity.

Following the modified *t*-test analysis [1,46], all significant deficits (p < 0.05) were further tested while controlling for the effects of covariates, in order to explore whether the dissociation remained when the effects of cofounding variables were removed.

**Fig. 1:**
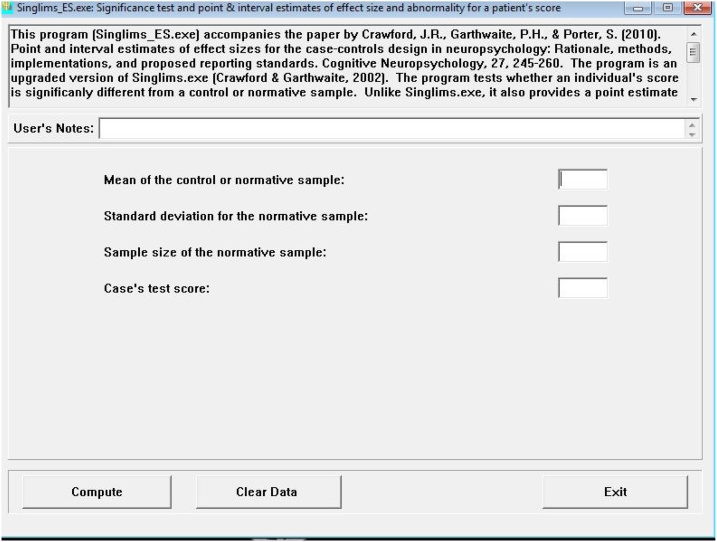
Screen shot of the program Singlims_ES.exe. The data requested must be introduced manually and the calculations are rendered immediately.

**Fig. 2:**
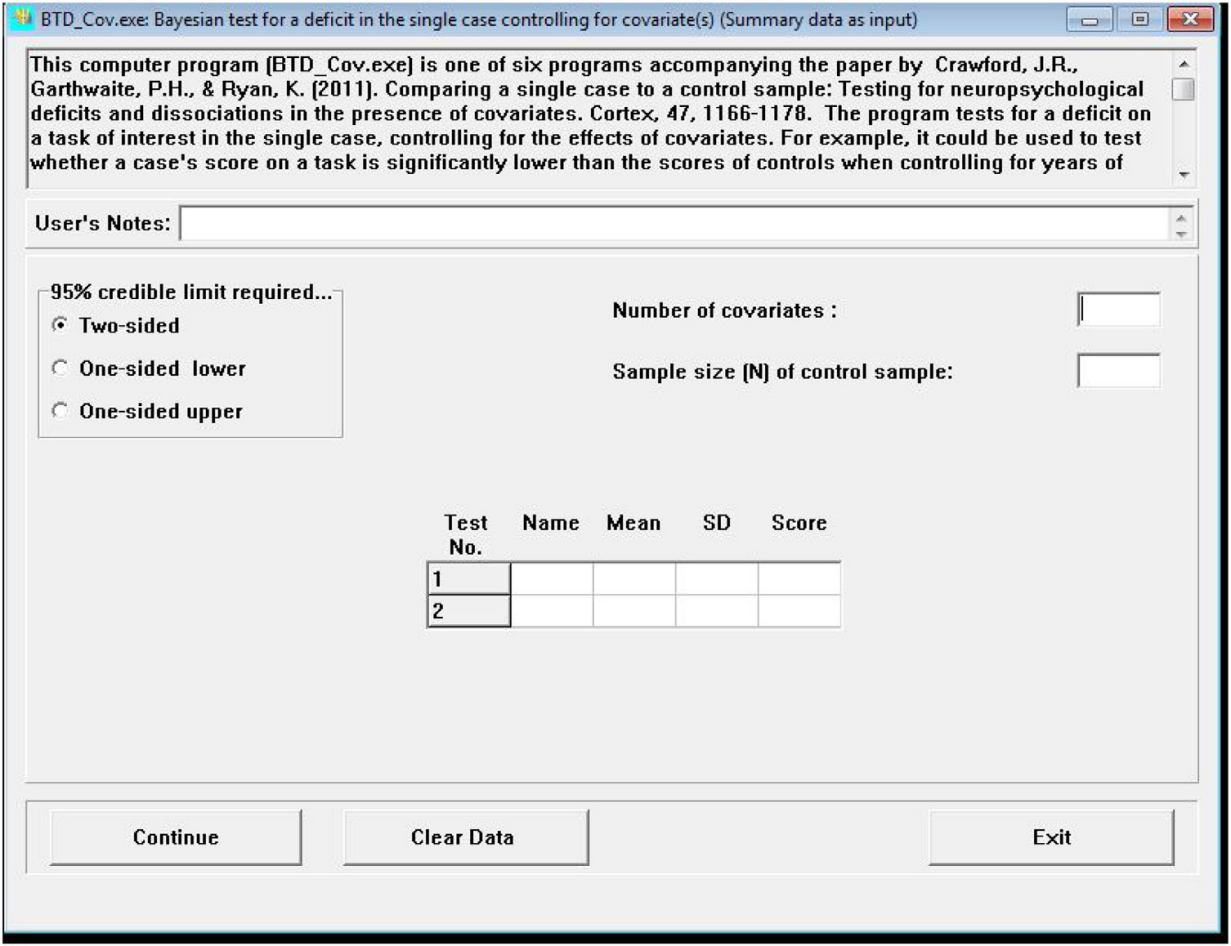
Screen shot of the program BTD_Cov.exe. The data requested must be introduced manually and the calculations are rendered immediately. The correlation matrix between the covariates and the test under investigation is necessary. This matrix expands according to the number of covariates introduced.

For these two *t-*tests (with and without consideration of covariates), a one-tailed test was used because there is a clear direction towards lower performance by the HDL2 cases in comparison to controls in the alternate hypothesis [49]. The program Singlims_ES.exe (Fig. 1) was used for the purpose of testing for deficits and dissociations [1,46,50], while the program BTD_Cov.exe (Fig. 2) was used to test if dissociations remained significant in the presence of selected covariates [7]. For these two *t-*tests (with and without consideration of covariates), a one-tailed test was used because there is a clear direction towards lower performance by the HDL2 cases in comparison to controls in the alternate hypothesis [49]. The program Singlims_ES.exe was used for the purpose of testing for deficits and dissociations [1,46,50], while the program BTD_Cov.exe was used to test if dissociation remained significant in the presence of selected covariates [7]. The data is summarised in tables following the authors’ guidelines [46] (Table 2).

**Table 2:** Crawford et al. [46] guidelines on the presentation of the data for the identification of deficits with and without controlling for covariates.

Test	Control Group (Ranges for age and years of education)			Case 1's scores	Significance Test^a^		Point estimate^b^	Estimated effect size (Z_cc_)	
	N	Mean	SD		*t*	*p*		Point	(95 % CI)
Task X									
Task Y									

n-1 degrees of freedom.

a Two tailed test.

b Estimated percentage of control population exhibiting a discrepancy more extreme than the case.

### Step 5: Analysis of dissociations

**Fig. 3:**
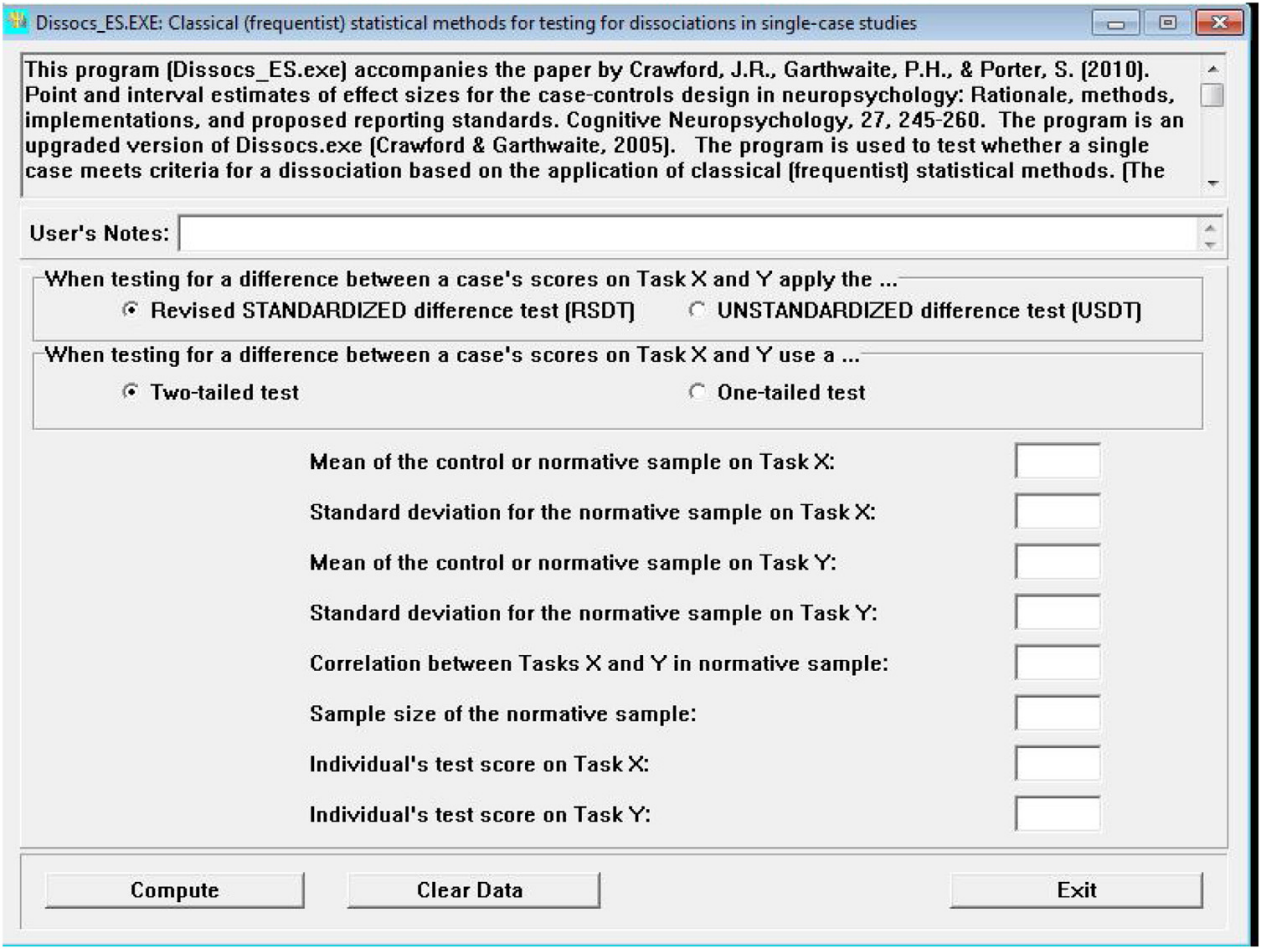
Screen shot of the program Singlims_ES.exe. The data requested must be introduced manually and the calculations are rendered immediately.

The intra-case variability was explored in order to establish whether the pattern of performance in the tasks meets the criteria of classical or strong dissociation [46,49]. The Revised Standardized Difference Test (RSDT) was used for testing the difference between the patient’s scores in two tasks, which is subsequently compared to the distribution of standardised differences from the control sample [49]. The program Dissoc_ES.exe (Fig. 3) was used and a two-tail analysis was selected because, although alternate hypotheses were available, they do not specify the exact direction in which the deficit will be displayed [49].

After comparing the HDL2 case to the control sample, it is possible to differentiate scores that are significantly below the norm (considered to represent a deficit) from those within normal limits. Subsequently, they can be compared to each other. If the intra-individual comparison yields a significant difference between a task (X) with a deficit and a task (Y) within normal limits, it is interpreted as a classical dissociation. If the scores in both tasks are significantly below the mean, and also significantly different from each other, it is interpreted as a strong dissociation [6]. The specific operational definitions provided by Crawford et al. [6] are Table 3:

- Classical dissociation: the patient’s score on a given test (A) is within normal limits while the patient’s score on a different test (B) has a deficit and the difference is significant using Crawford et al.’s [8] method and a two-tailed test (p < 0.05).
- Strong dissociation: the patient’s score on two given tests (A and B) present with a deficit, but one score is significantly lower than the other using Crawford et al.’s [8] method and a two-tailed test (p < 0.05).

**Table 3:** Crawford et al. [46] guidelines on the presentation of the data for the identification of classical and strong dissociations.

		Control Group			Case 1′s scores	Significance Test^a^		Estimated percentage of the control population obtaining a lower score than the case		Estimated effect size (Z_cc_)	
		(Ranges for age and years of education)									
Tests		N	Mean	SD		*t*	*p*	Point	(95 % CI)	Point	(95 % CI)
Task X											
Task Y											

n-1 degrees of freedom.

^b^Estimated percentage of control population exhibiting a discrepancy more extreme than the case.

Note: This level analysis provides significance tests, point and interval estimates of the percentage of pairs of controls that will exhibit a larger abnormality (or discrepancy), and the point and interval of the effect sizes [7,46].

a Two tailed test.

In progressive conditions, such as HDL2, the relevance of this analysis is accentuated because the possibility of a generalised decline introduces the risk of having trivial differences between performances that may be misinterpreted [46]. With this test, it is possible to identify those functions that are specifically affected for each case, thus allowing for the identification of dissociations and introducing the issue of the severity of deficit for each individual, who is at a particular stage of the disease and therefore, at a specific moment of neuropathological progression. Therefore, the tasks yielding a strong dissociation would be interpreted as more severely affected than those displaying a classical dissociation.

## Footnotes

1 This study adheres to the definitions of terms proposed by Krause et al. [20]: “Mixed ancestry individuals, in South Africa, are those whose gene pool is derived from one or more of the indigenous African populations (San, Khoikhoi or Bantu speaking), European immigrants from western Europe and/or slaves and indentured labourers from Madagascar, the Malaysian archipelago and India”. “Black individuals are those whose gene pool is derived from one or more of the indigenous Bantu-speaking African populations” (p. 1).

2 Raw scores were used in all data analyses in order to avoid superimposing specific distributions attached to adjusted scores and to allow for more variability in the scores, especially because some of the measurements have a narrow range and ceiling effects.
