# Supplementary figures and images for: Single case-control design for the study of the neuropsychological deficits and dissociations in Huntington’s disease-like 2
Source: MethodsX. 2020 Jan 10;7:100782. doi: 10.1016/j.mex.2020.100782 (PMC6995251; doi:10.1016/j.mex.2020.100782)

Supplementary Data File 1

Histograms for all Variables for Clinical and Control Groups

| 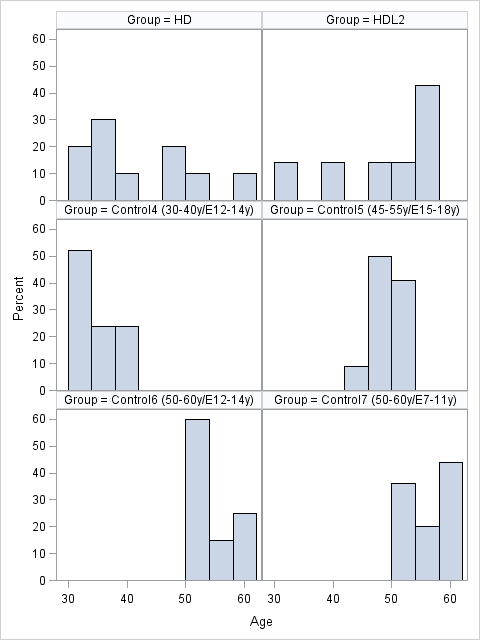 | 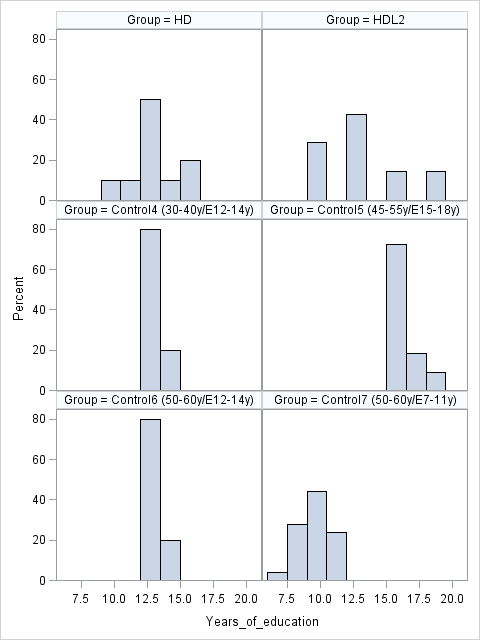 |
| --- | --- |
| 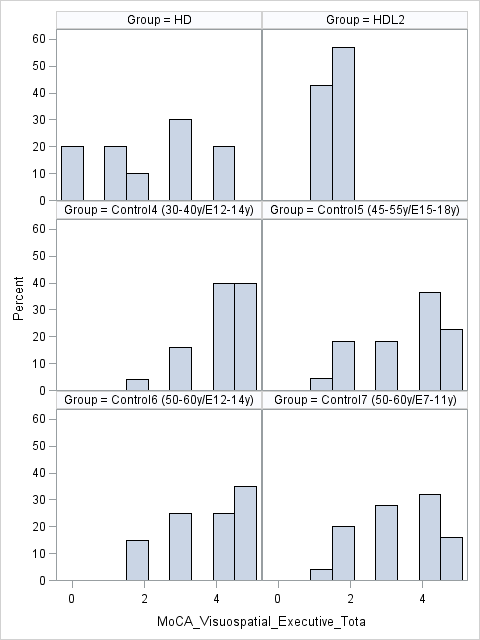 | 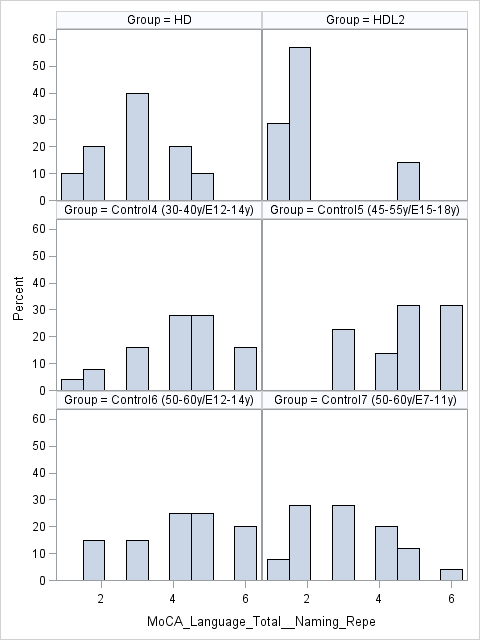 |

| 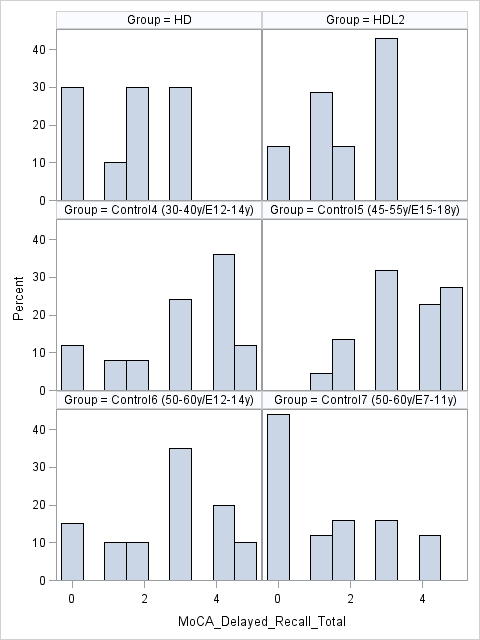 | 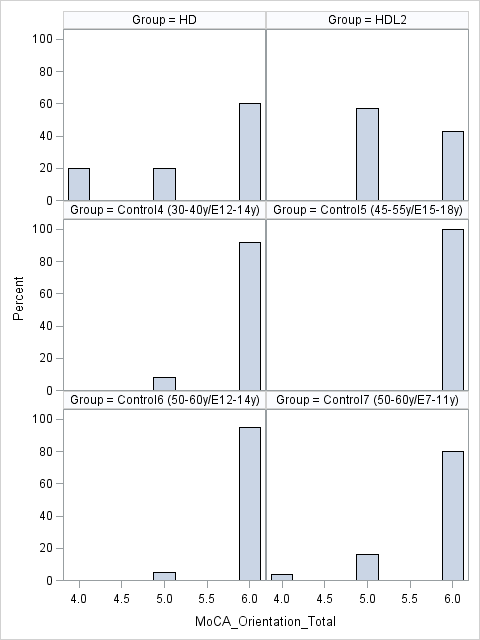 |
| --- | --- |
| 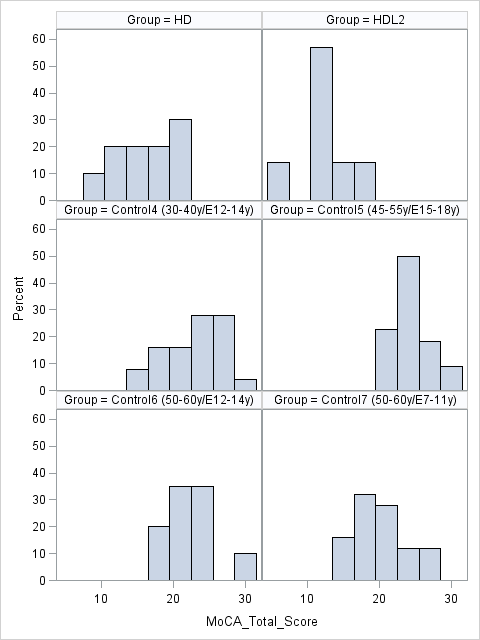 | 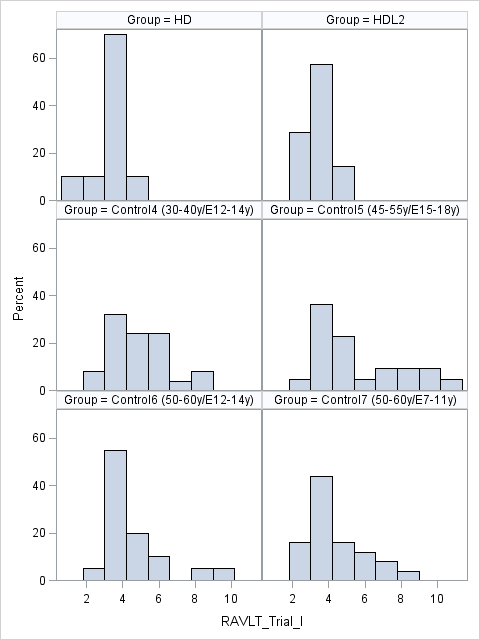 |

| 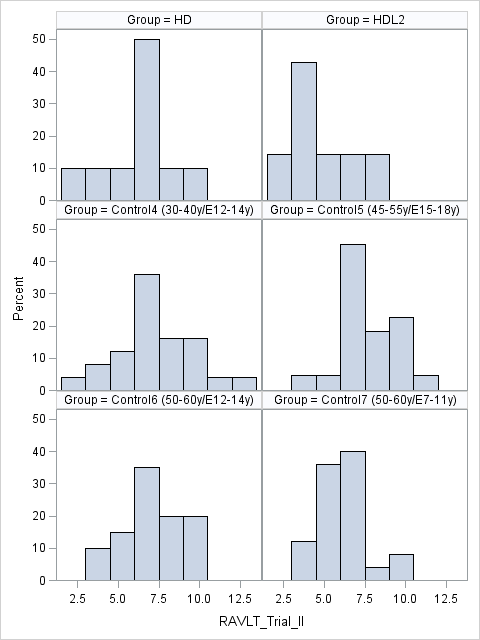 | 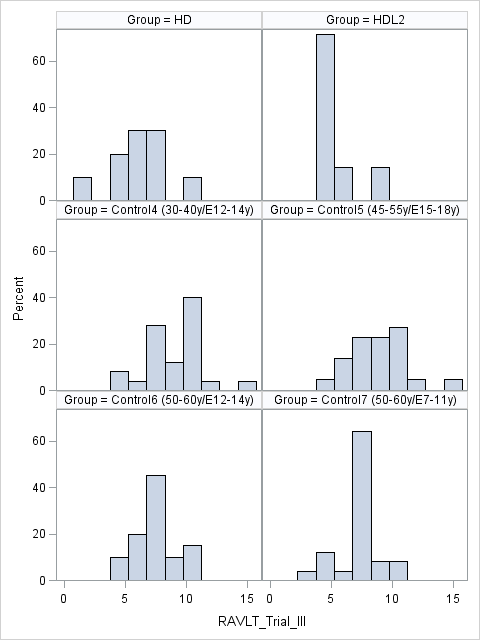 |
| --- | --- |
| 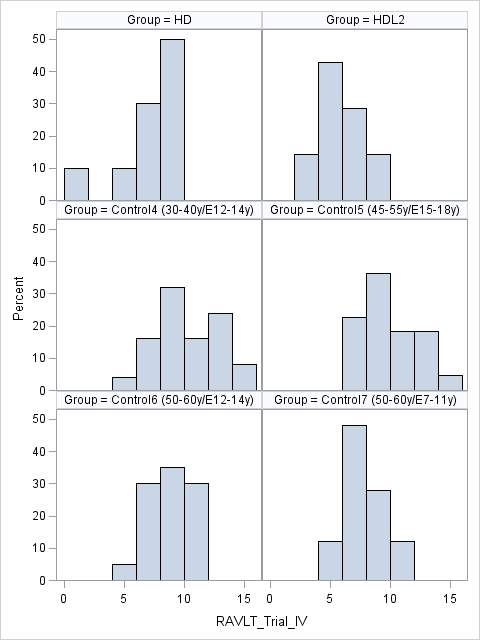 | 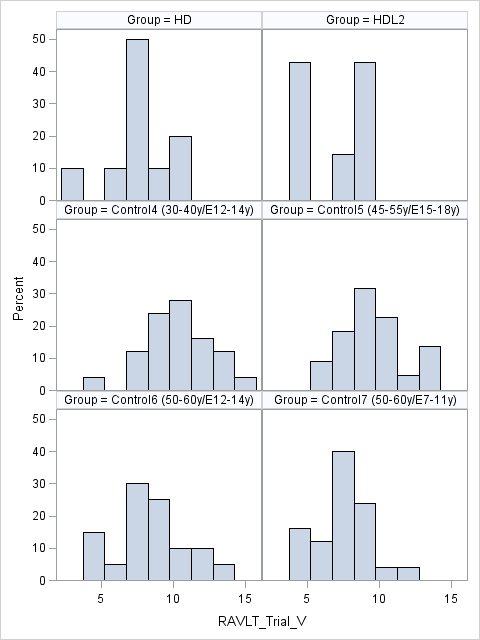 |

| 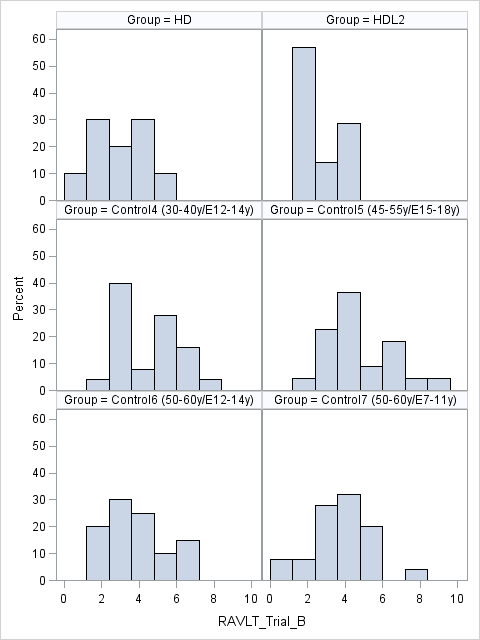 | 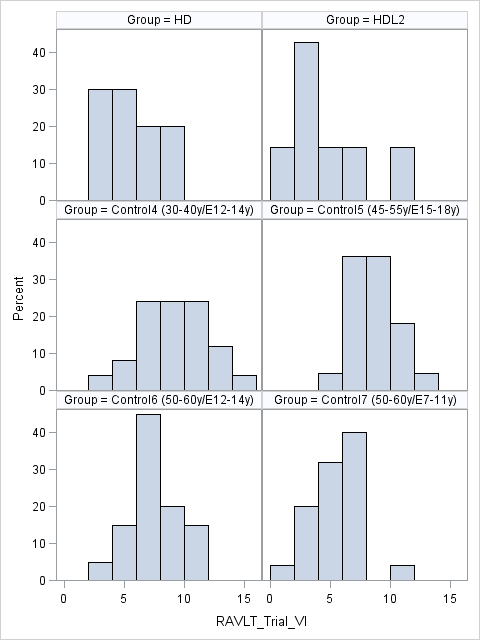 |
| --- | --- |
| 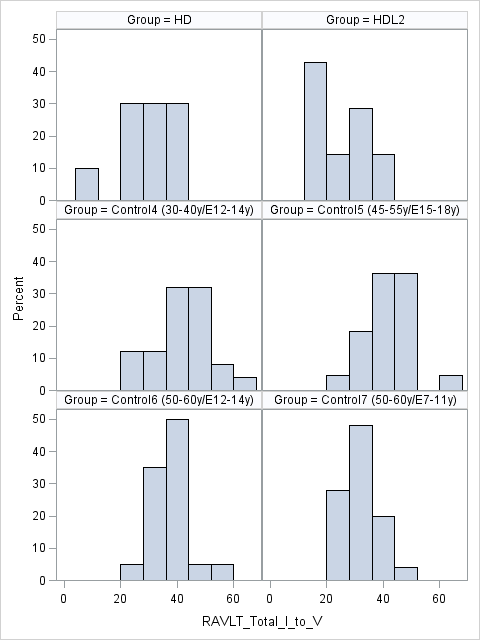 | 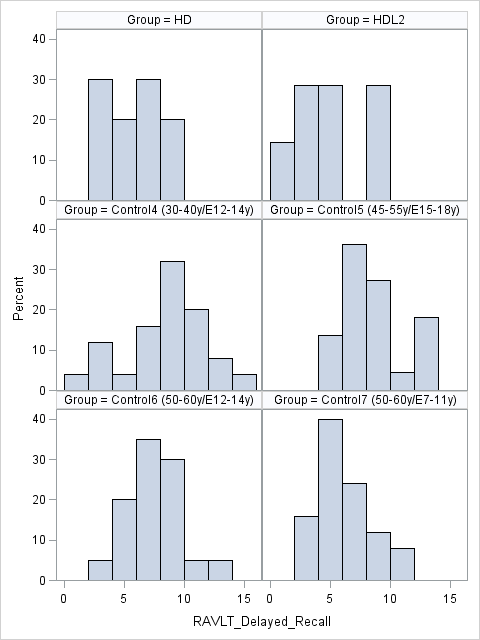 |
| 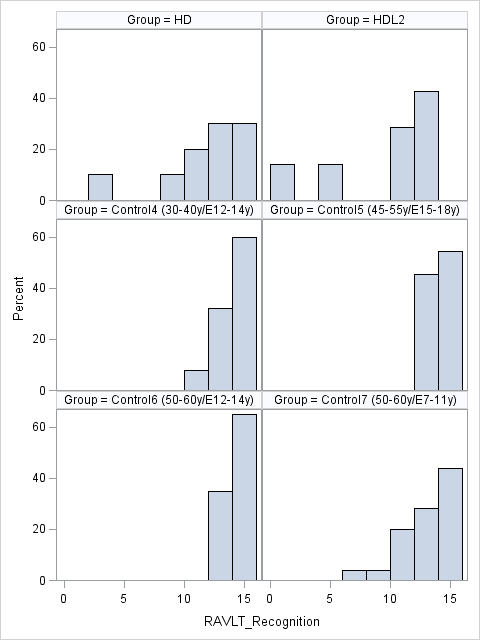 | 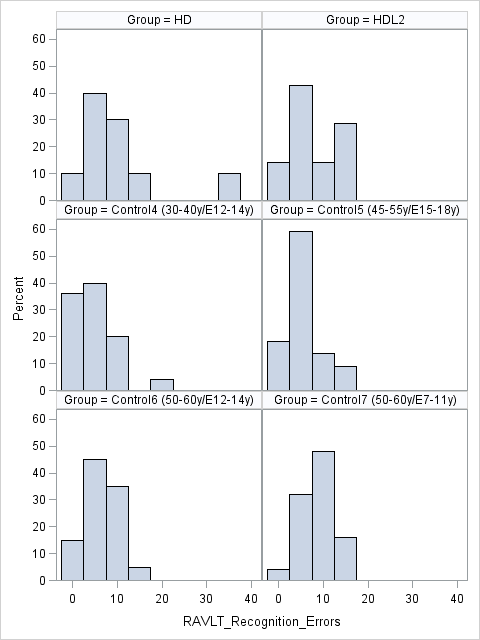 |
| 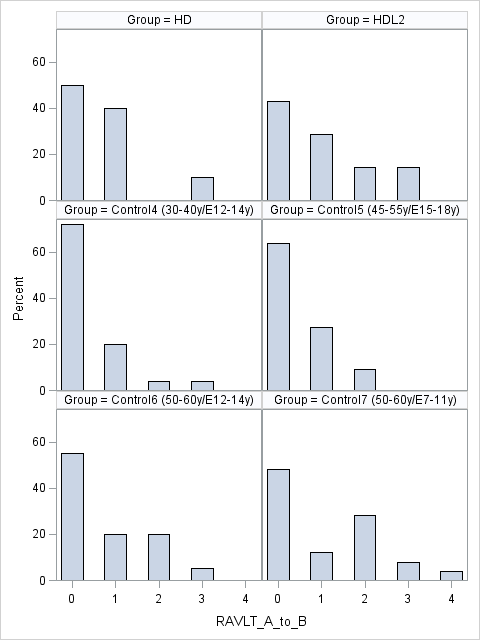 | 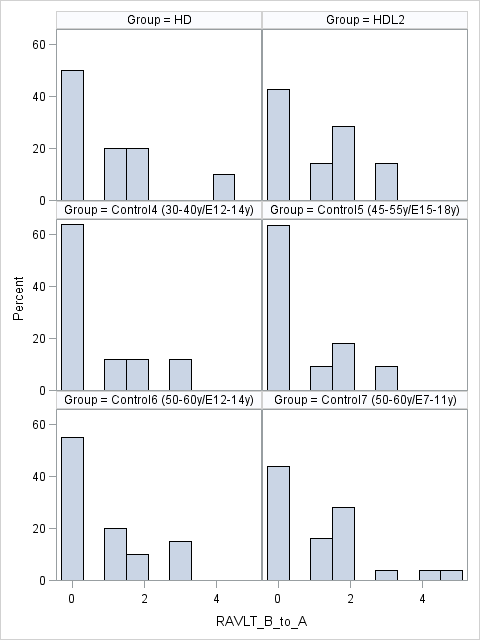 |

| 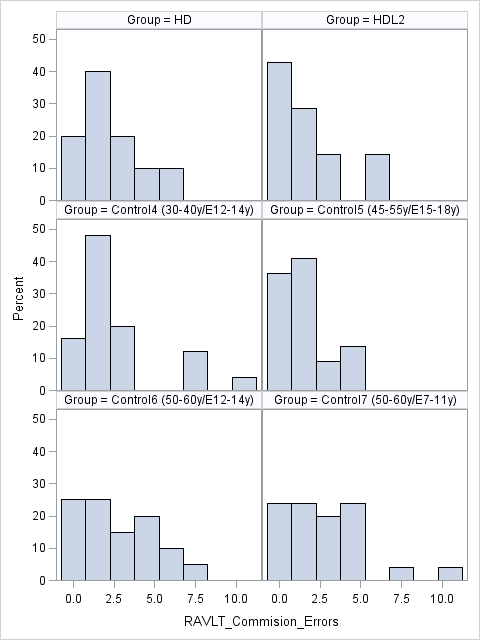 | 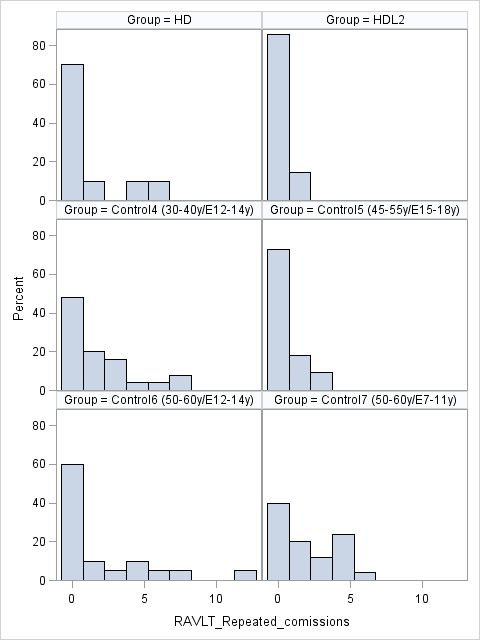 |
| --- | --- |
| 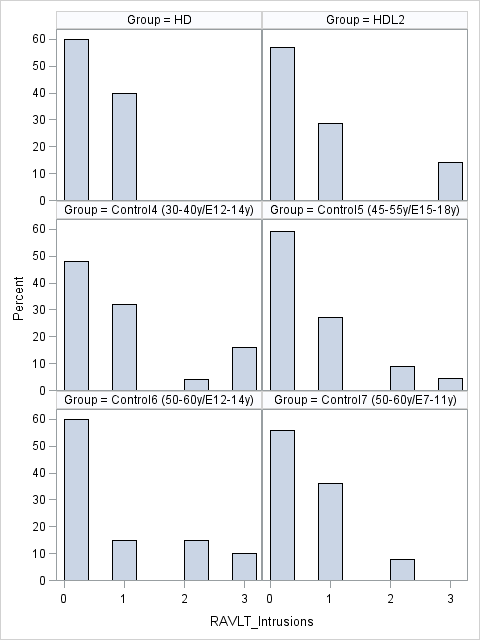 | 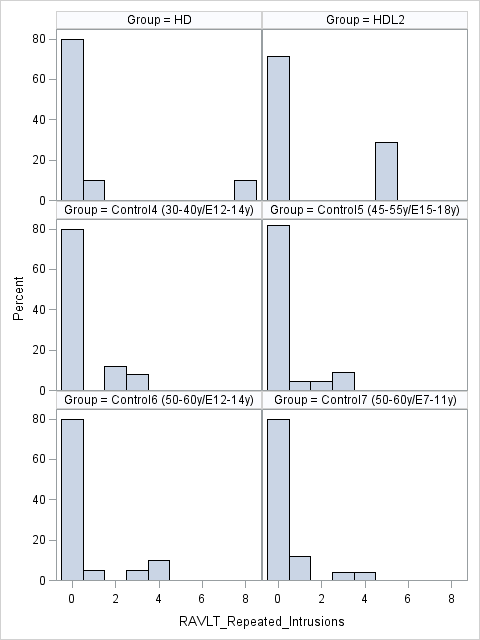 |

| 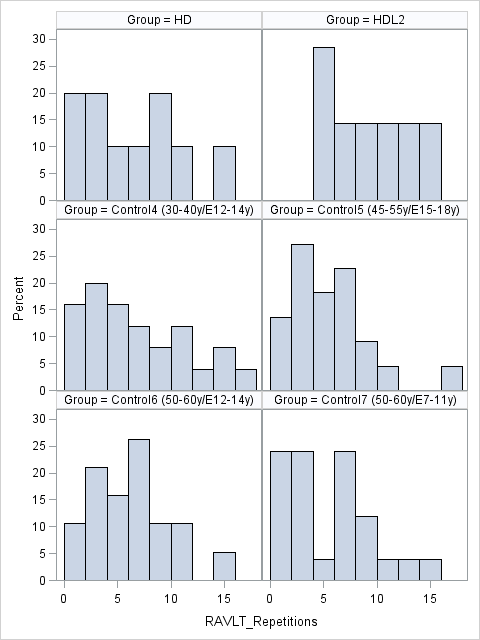 | 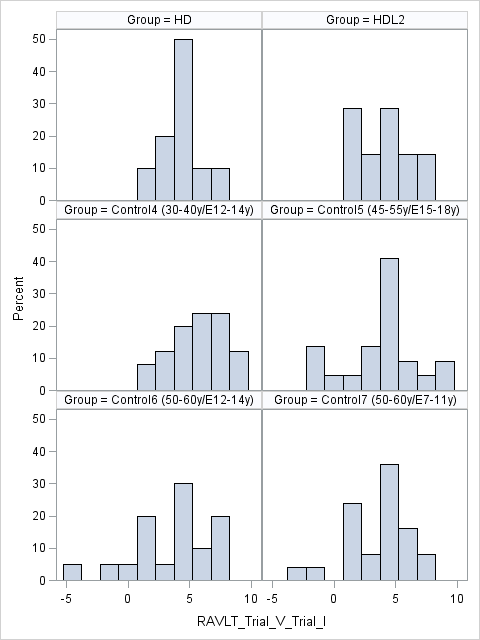 |
| --- | --- |
| 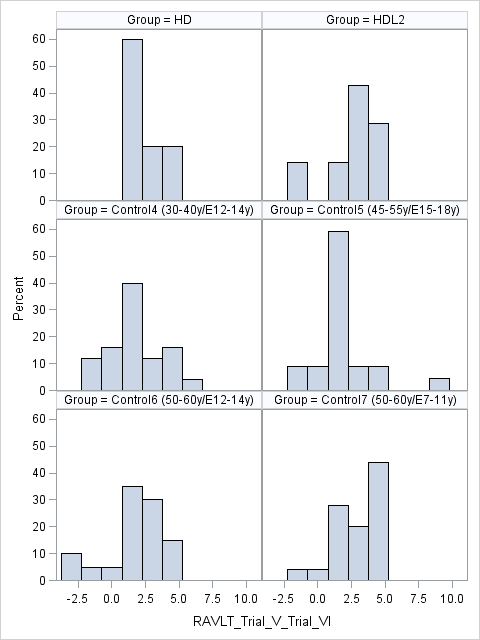 | 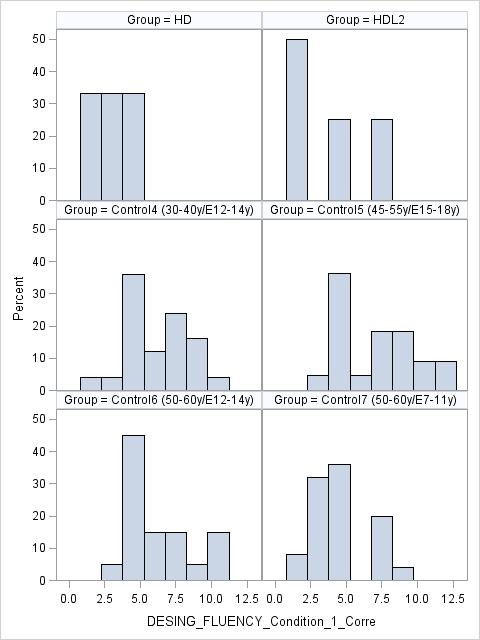 |

| 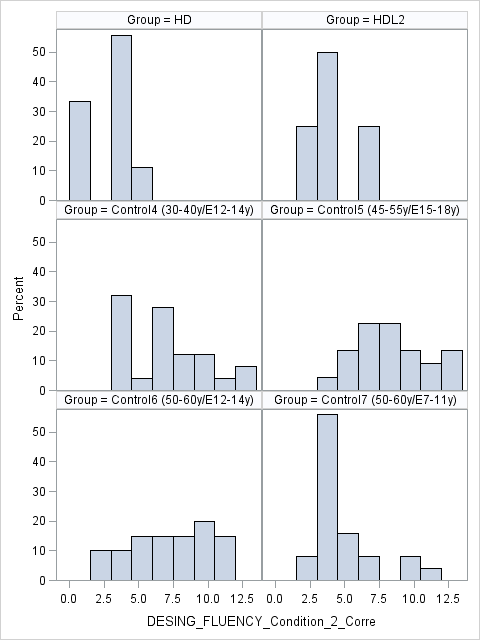 | 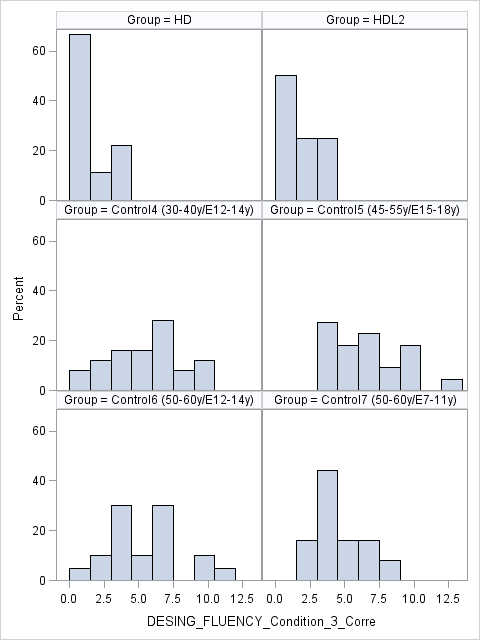 |
| --- | --- |
| 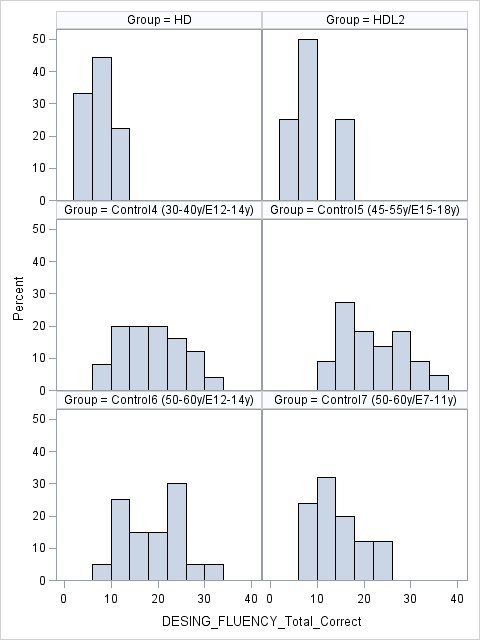 | 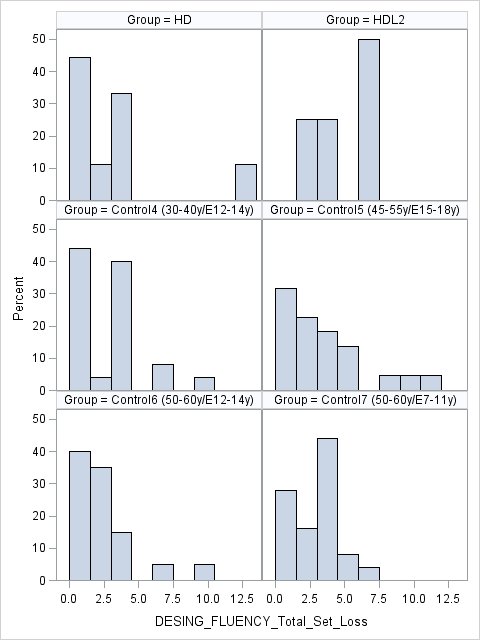 |

| 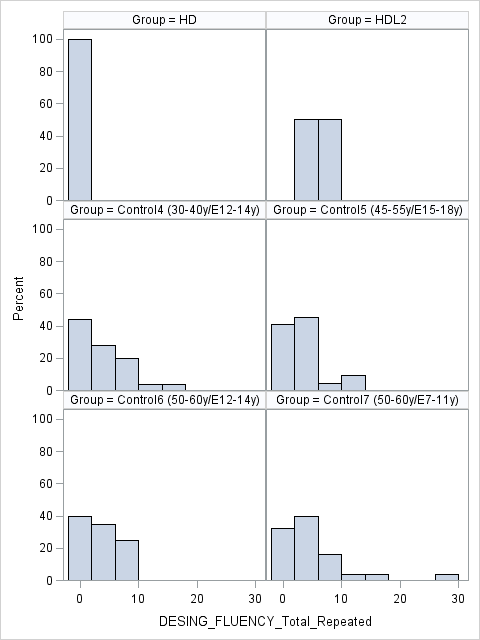 | 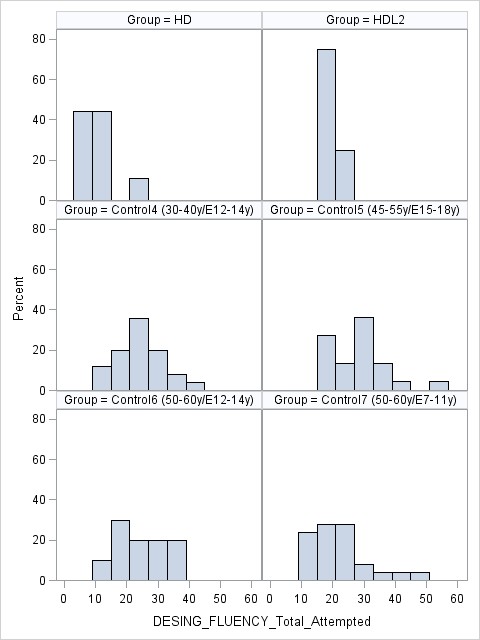 |
| --- | --- |
| 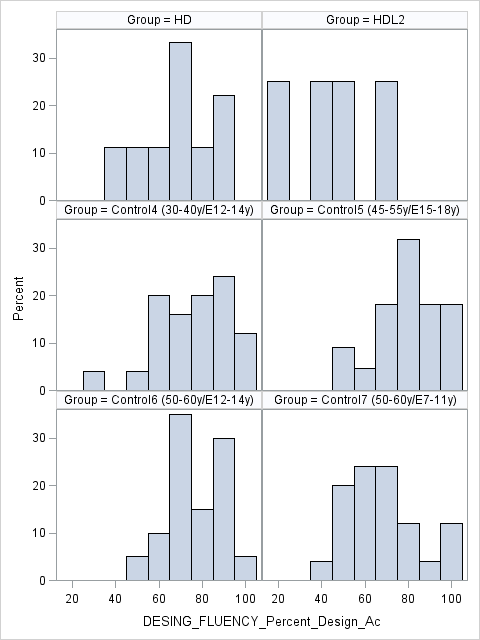 | 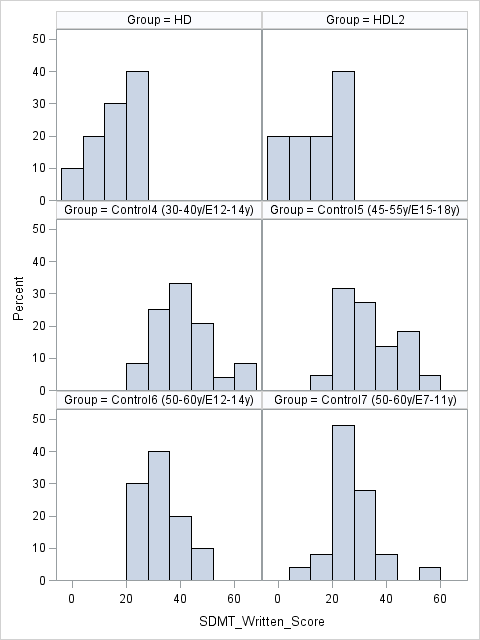 |

| 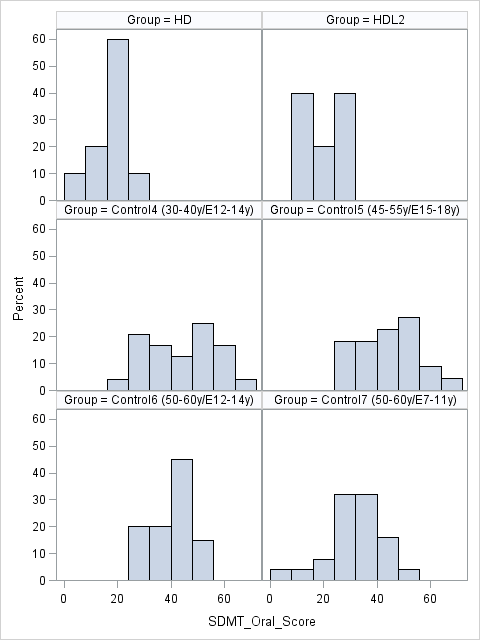 | 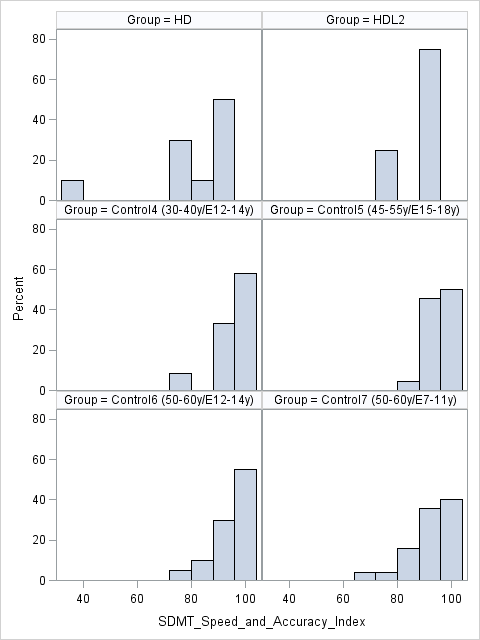 |
| --- | --- |
| 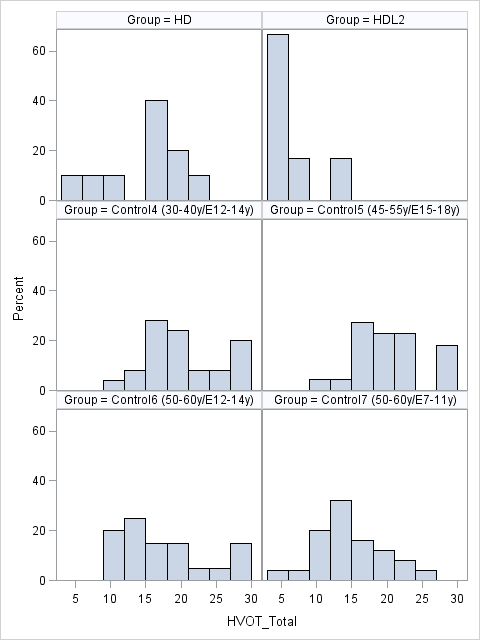 | 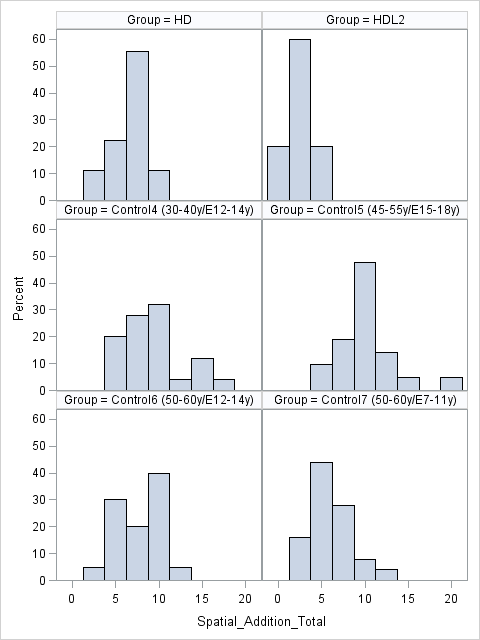 |

| 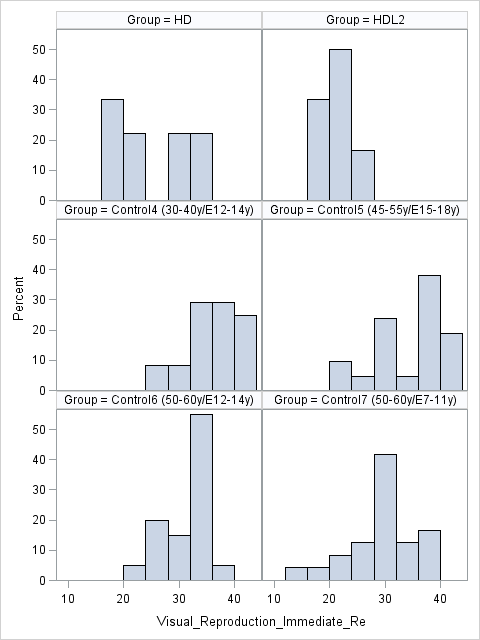 | 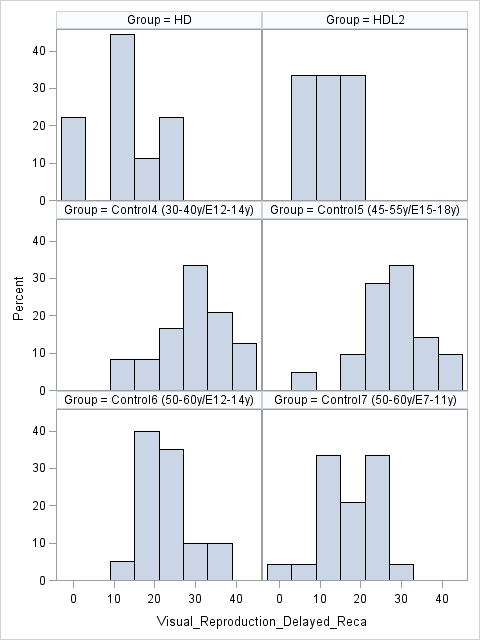 |
| --- | --- |
| 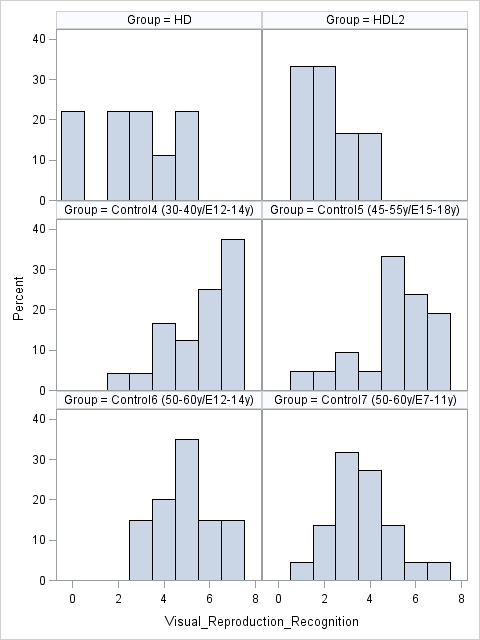 | 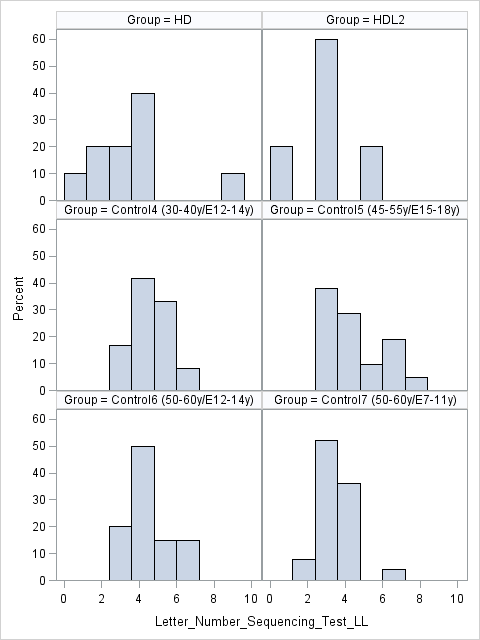 |
|  |  |
| 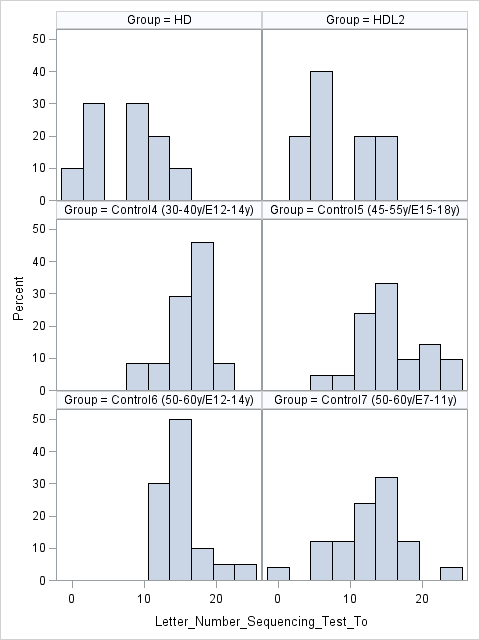 | 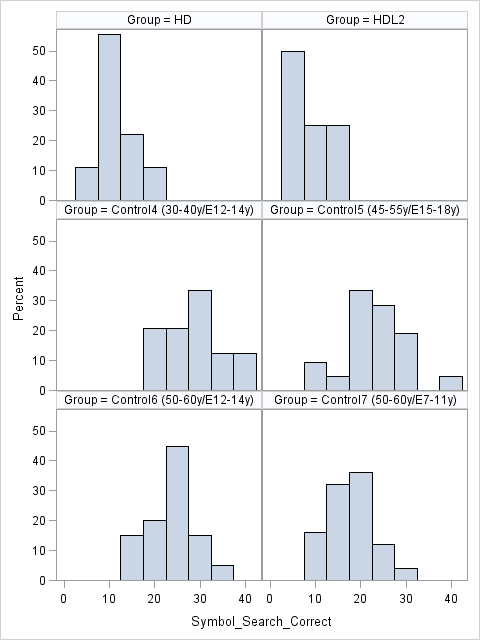 |
| 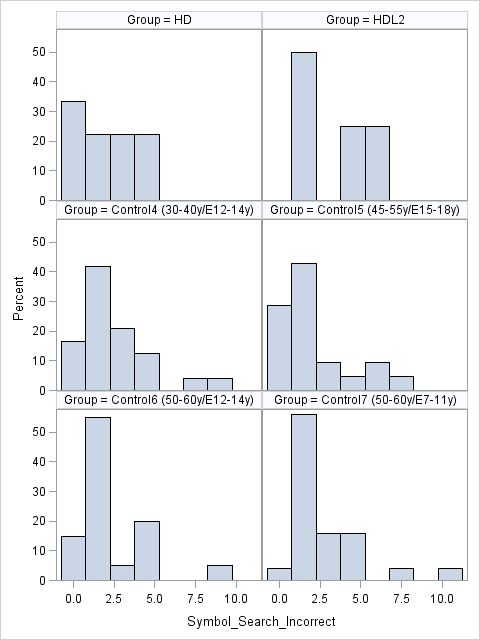 | 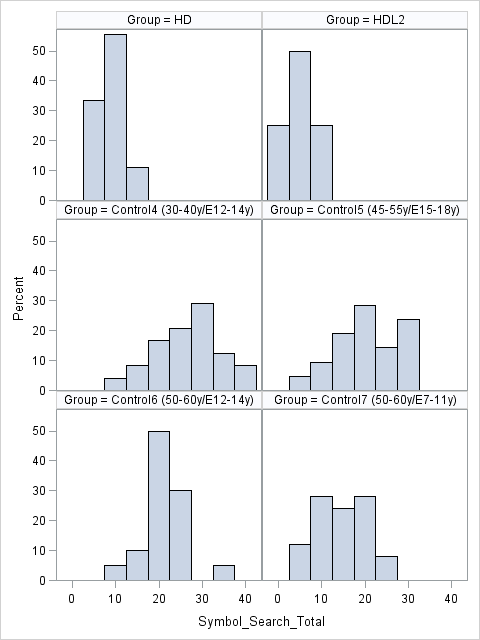 |

| 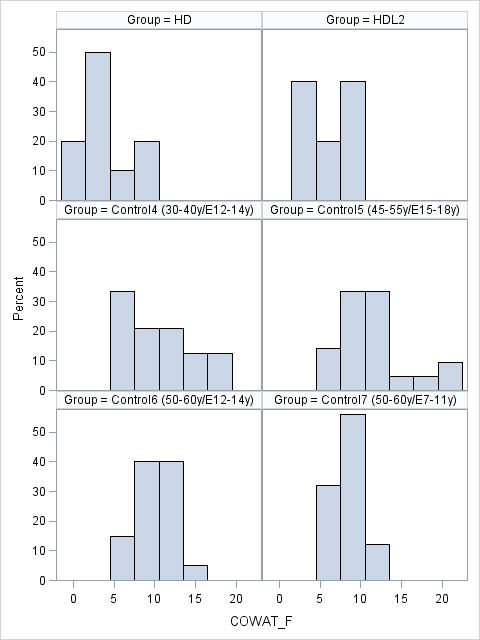 | 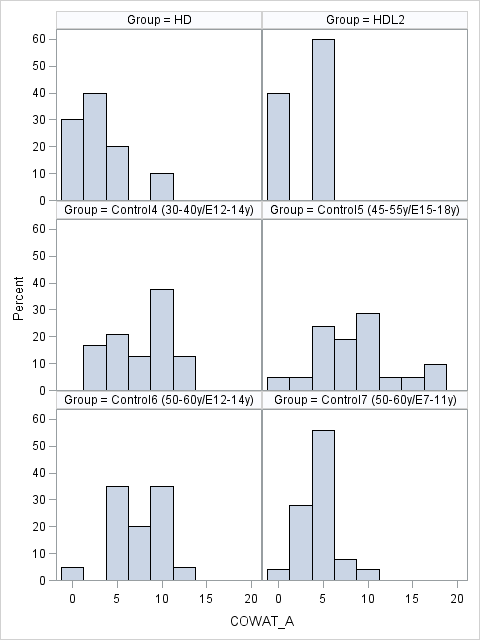 |
| --- | --- |
| 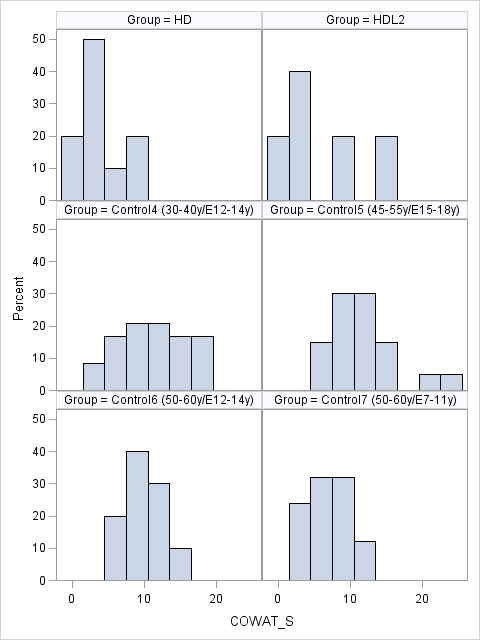 | 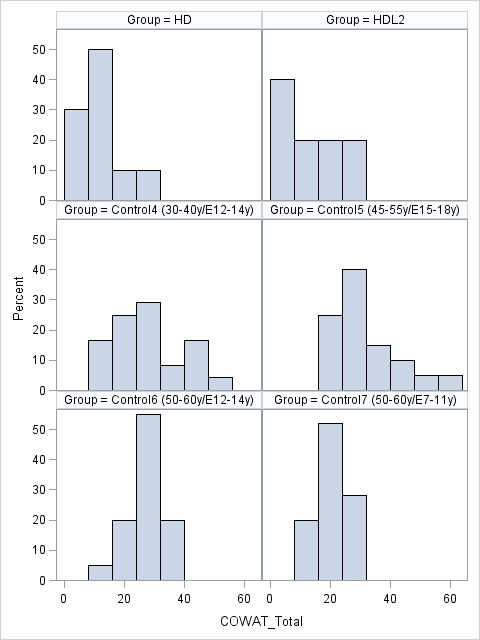 |
| 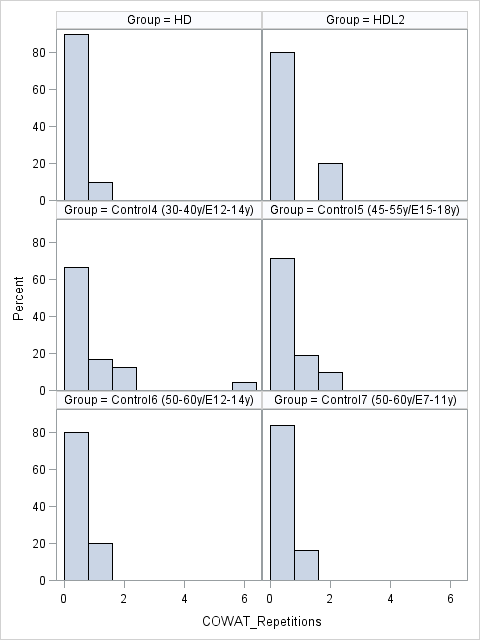 | 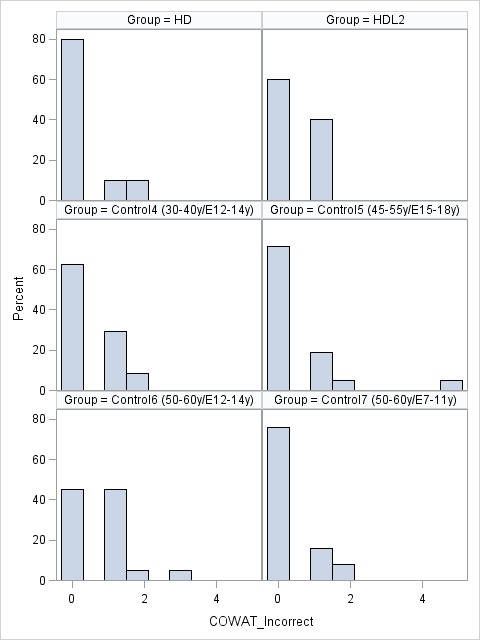 |
| 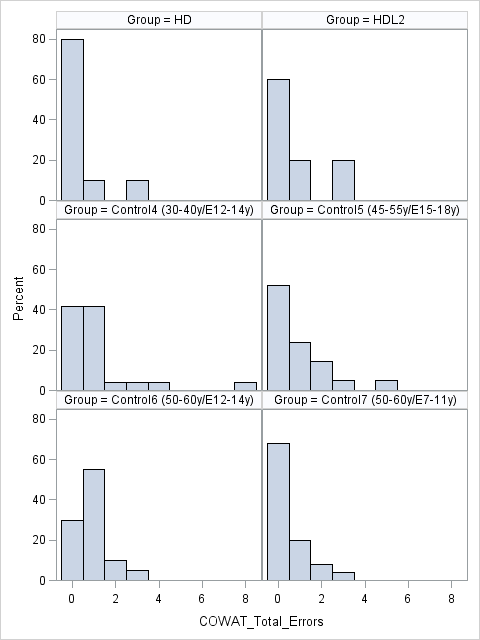 | 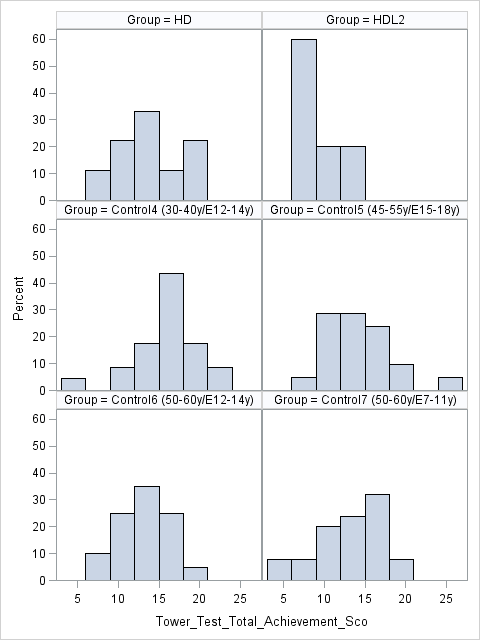 |

| 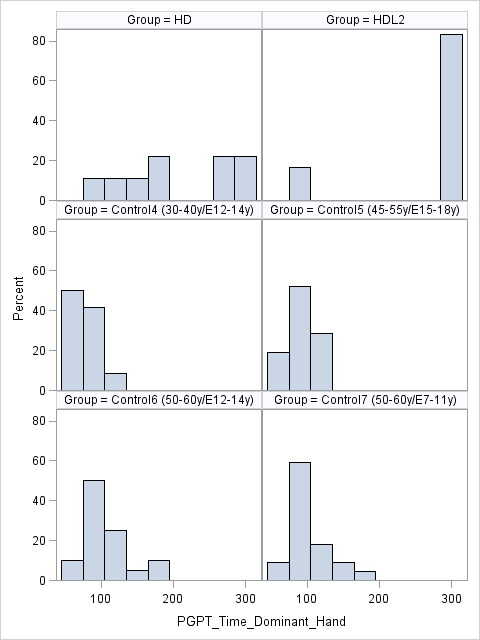 | 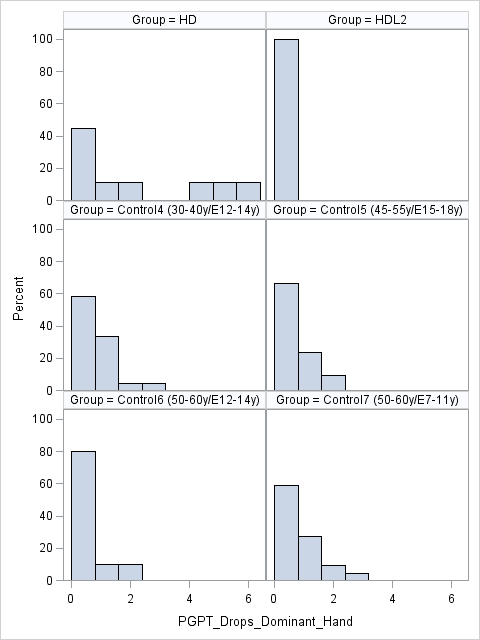 |
| --- | --- |
| 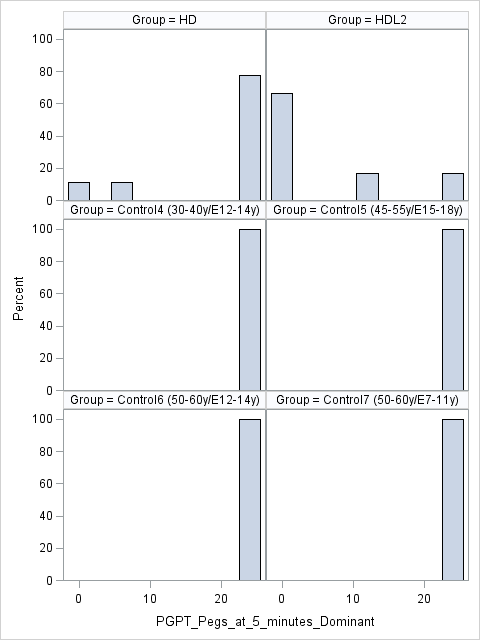 | 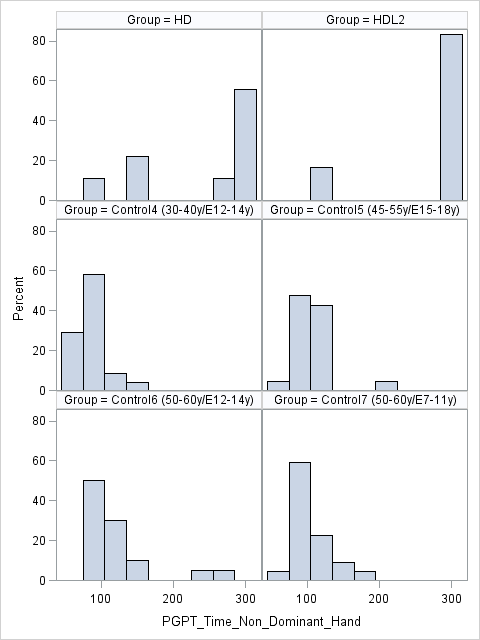 |

| 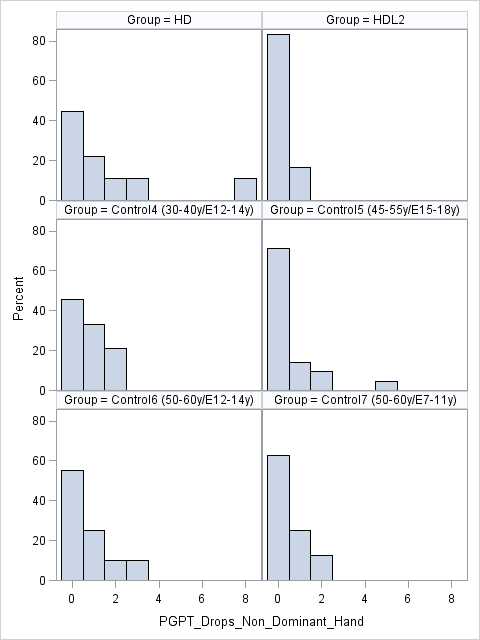 | 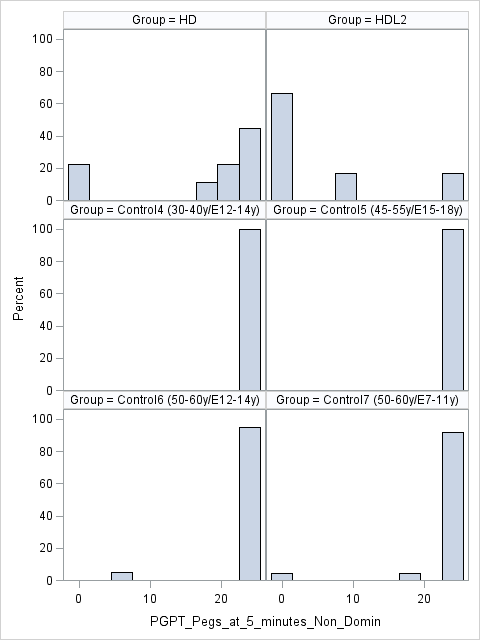 |
| --- | --- |
| 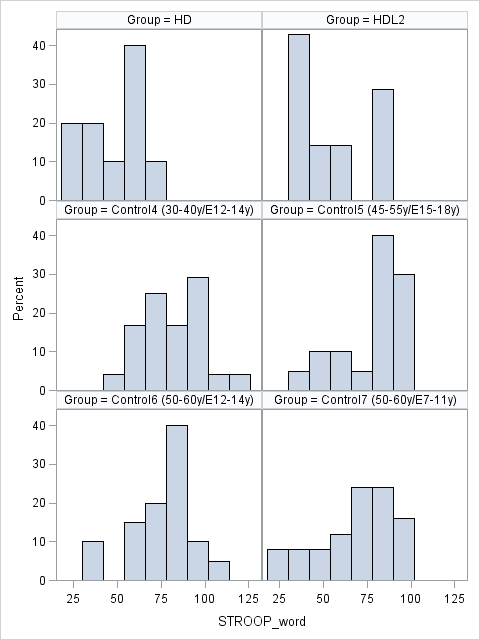 | 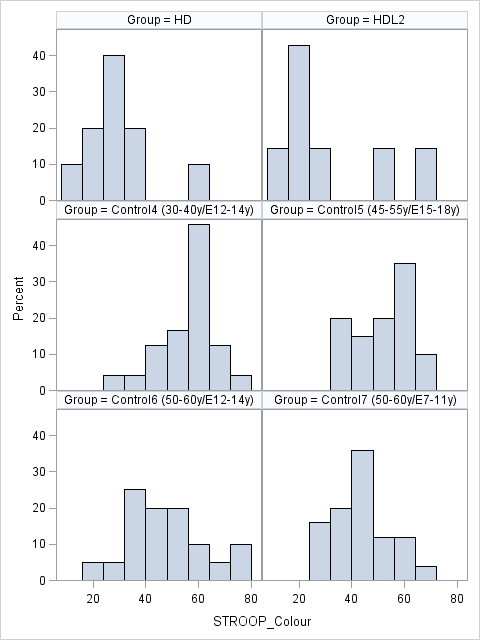 |

| 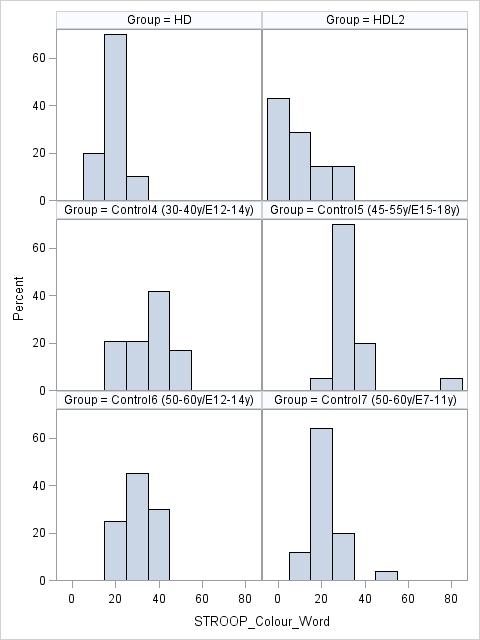 |  |
| --- | --- |
|  |  |

Supplement: Supplementary file 1 [file mmc1.docx]
